# Significance of preoperative left ventricular ejection fraction in 5-year outcome after isolated CABG

**DOI:** 10.1186/s13019-021-01732-3

**Published:** 2021-12-27

**Authors:** Aida Fallahzadeh, Ali Sheikhy, Ali Ajam, Saeed Sadeghian, Mina Pashang, Mahmoud Shirzad, Jamshid Bagheri, Soheil Mansourian, Shahram Momtahen, Kaveh Hosseini

**Affiliations:** 1 Research Department, Tehran Heart Center, Tehran University of Medical Sciences, Tehran, Iran; 2 Students’ Scientific Research Center (SSRC), Tehran University of Medical Sciences, Tehran, Iran; 3 Department of Cardiology, Tehran Heart Center, Tehran University of Medical Sciences, North Karegar Ave, PO Box: 1411713138, Tehran, Iran; 4 Tehran Heart Center, Tehran University of Medical Sciences, Tehran, Iran; 5 Department of Surgery, Tehran Heart Center, Tehran University of Medical Sciences, Tehran, Iran

**Keywords:** Coronary artery bypass graft (CABG), Left ventricular ejection fraction (LVEF), Mortality

## Abstract

**Background:**

Pre-operative ejection fraction (EF) and comorbidities affect post-op outcomes. We aimed to compare the mortality and adverse events of patients with different baseline EF and also to evaluate the distribution of comorbidities in each EF group.

**Methods:**

A total of 20,937 patients who underwent isolated coronary artery bypass graft (CABG) surgery from January 2006 to December 2016 was included. Patients were divided into three groups based on their pre-operative left ventricular EF as follows; (1) Normal: EF ≥ 50%; (2) Mild to moderately reduced: 50% < EF ≤ 35%; and (3) Severely reduced: EF < 35%. The backward elimination method was considered for multivariate Cox-regression analysis to locate predictors of mortality and non-fatal cerebro-cardiovascular events (CCVEs). The median follow-up time was 5.61 [3.12–8.0] years.

**Results:**

The mean age in the total population was 60.94 ± 9.51 years and 73.6% of the total population was male. Diabetes mellitus was the common risk factor of mortality and CCVE in all EF groups. Impaired renal function (GFR < 60 ml/min) was associated with a higher risk of mortality after CABG regardless of EF level. The median 5-year mortality rate in patients with normal EF, mild-moderately reduced EF and severely reduced EF were 9.5%, 12.8%, and 22.7% respectively (*P* < 0.001). Although the trend of CCVEs was higher in severe left ventricle (LV) dysfunction, it was not statistically significant (*p* = 0.071).

**Conclusion:**

Patients with severely reduced EF are at higher risk of mortality after CABG compared to those with higher EF levels; however, the rate of CCVEs may not be necessarily higher after adjustment for multiple pre-operative comorbidities.

**Supplementary Information:**

The online version contains supplementary material available at 10.1186/s13019-021-01732-3.

## Introduction

Coronary artery disease (CAD) is the most common type of heart disease and the third leading cause of death in both women and men worldwide [1]. Coronary artery bypass graft (CABG) is the most performed procedure in patients with multivessel coronary artery diseases [2]. Indeed, several perioperative risk factors have been reported to affect outcomes after CABG [3].

Traditional predictors of adverse outcomes after CABG are older age, female gender, diabetes mellitus, hypertension, chronic obstructive pulmonary disease (COPD), renal impairment, left main stem disease, and low left ventricular ejection fraction (LVEF) [4]. Therefore, identifying the predictors that may be associated with worse outcomes after CABG, plays an important role in making a clinical decision and patient selection [5].

Low LVEF is an important predictor of mortality and morbidity after CABG; however, CABG is the treatment of choice in patients with impaired LVEF and is associated with better survival compared to medical therapy alone [6–8]. CABG in such patients is associated with higher postoperative morbidity and mortality compared to those with normal left ventricular function [9, 10]. However, patients with impaired LVEF have higher preoperative comorbid conditions [11]. Patients with different ejection fraction (EF) levels may have different comorbidities which will affect the postoperative outcomes. Several studies evaluated the in-hospital survival and predictors of early outcomes after CABG in patients with low LVEF [3, 11, 12]; however, few studies focused on comparing the predictors in different EF groups (normal left ventricular [LV] function > 50%, mild to moderate LV dysfunction 35–50% and severe LV dysfunction < 35%) and the mid-term and long-term outcomes. Decreased EF may be related to thromboembolic events through stasis of blood flow, endothelial injury, and hypercoagulability (Virchow triad) [13] and the severity of CAD may be related to the severity of atherosclerotic burden in carotid arteries [14]; thus, the purpose of this study was to identify and compare independent predictors of mortality and cerebro-cardiovascular events (CCVEs) in three pre-operative LVEF levels.

## Material and method

### Study cohort

**Fig. 1:**
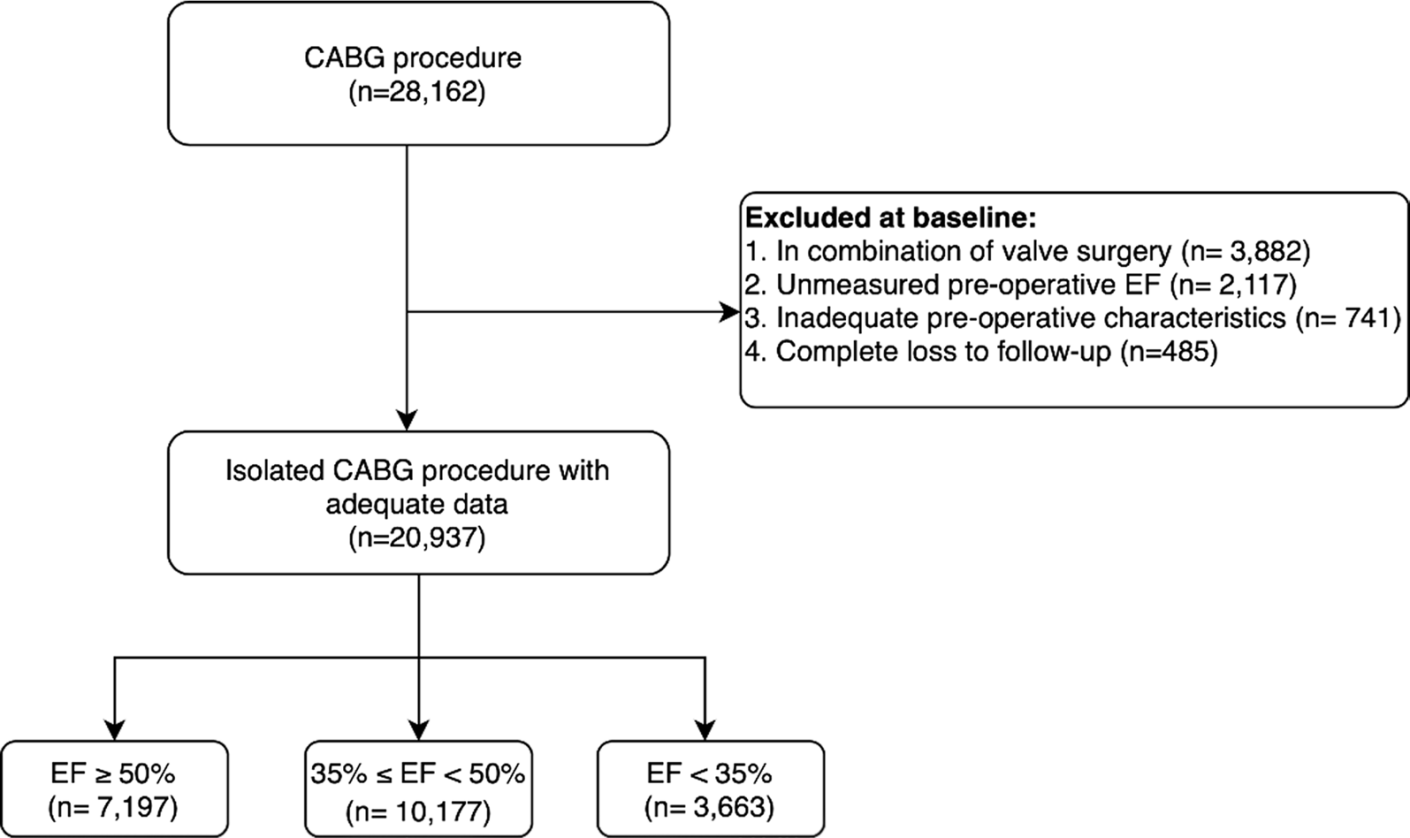
Study cohort flow-chart

This study is a retrospective registry-based cohort study that was conducted in the clinical registry of Tehran Heart Center (THC) [15]. All data was recorded prospectively at the time of admission. We reported this study according to the Strengthening the Reporting of Observational Studies in Epidemiology (STROBE) statement. This study included patients who underwent CABG surgery from January 2006 to December 2016 and were prospectively followed until 2020. Patients with a lack of sufficient data and those with severe mitral valve regurgitation (MR) (due to overestimation of EF in severe MR) were eliminated from the study. Inclusion criteria were as follows:1) Surgical revascularization criteria for ischemic heart disease and 2) Isolated CABG excluding valve surgeries. Conclusively, 20,937 patients were recruited in the final analysis (Fig. 1). Patients were divided into three groups based on their pre-operative LVEF as follows; (1) EF ≥ 50%; (2) 50% < EF ≤ 35%; and (3) EF < 35%. The study was approved by the ethical board of THC (IR-THC-13799) and involving human data was under the guidelines of the Declaration of Helsinki. This study didn’t meet the criteria for an informed consent form.

### Definition of variables

The following data were included for analysis: Demographic characteristics, graft numbers, comorbidities, preoperative risk factors, and urgency of surgery. Each variable is defined in the Additional file 1.

### Surgical technique

Left and right internal mammillary arteries (LIMA and RIMA) and saphenous vein grafts (SVG) were harvested by the “No-touch” technique. In the routine procedure, LIMA was used for the left ascending artery (LAD) and SVG for the right coronary, left circumflex, and diagonal artery, furthermore the grafting conduits selection was based on the surgeon’s preference concerning.

For the on-pump CABG procedure, to achieve cardiopulmonary bypass (CPB), a single right atrium and aortic cannulation were made, also, to conserve activated clotting time (ACT) at ≥ 480 s, Heparin was used. Anterograde cold blood cardioplegia was conducted through the surgery. At the end of the surgery, Protamine sulfate was prescribed to neutralize the Heparin. To avoid hypothermia-induced arrhythmia, the patients’ systemic temperature was sustained at 36 °C.

For the off-pump CABG procedure, a carbon dioxide blower (Medtronic Inc., Minneapolis, MN) was used for better visualization of the operative field and anastomosis cites. ACT reached ≥ 350 s by using Heparin. 6‐0 monofilament sutures were made for the proximal anastomoses to the aorta, whereas 8–0 monofilament sutures were used for distal anastomosis.

### Follow up and study endpoint

The patients' follow-up protocol was as 4th, 6th, and 12th month of surgery and annually after the last visit through attending visits at the post-op clinics. Telephone interviews were made for individuals who were unable to attend the clinics.

The primary endpoints were mid-term (median 5 years) all-cause mortality and non-fatal CCVEs occurrence (comprising of non-fatal acute coronary syndromes [ACS], non-fatal stroke or transient ischemic attack [TIA], and repeat coronary revascularization via percutaneous coronary intervention [PCI]or redo-CABG).

### Statistical analysis

Descriptive statistics were used to describe baseline characteristics, subsequently, categorical variables were described as absolute and frequencies, and continuous variables were reported as mean and standard deviation or median and interquartile range according to their distribution. The “Chi-square goodness of fit” was used to compare categorical variables. Normally and non-normally distributed continuous variables were compared using one-way ANOVA (Analysis of variance) and Kruskal–Wallis test, respectively.

The univariate effect of covariates on mortality and non-fatal CCVEs was assessed by the univariate Cox regression model and reported as hazards ratio (HR) with 95% confidence intervals. Covariates with P values less than 0.1 in the univariate Cox regression analyses were entered multivariate Cox-regression analysis. The backward elimination method was considered for multivariate Cox-regression analysis to locate predictors of mortality and non-fatal CCVEs. The Proportional hazard assumption was tested through a graphical assessment based on the scaled Schoenfeld residuals for each final model’s variable. Proportional hazard assumption was met for each variable (Additional file 1: Figure S1).

All statistical analyses were conducted applying Stata Statistical Software, release 14 (College Station, TX: StataCorp LP) and R version 4.0.3 [16]. Besides, we used several packages in R: "survival" (package for survival analysis in R) [17], "survminer" (drawing survival curves) [18], and “ggplot2” [19].

## Results

### Baseline characteristics

From January 2006 to December 2016, a total of 20,937 patients who underwent isolated CABG procedures, were included. The median follow-up time was 5.61 [3.12–8.0] years. The mean age in the total population was 60.94 ± 9.51 years also, 73.6% of the total population was male.

In patients with EF ≥ 50%, the mean of age was 60.75 ± 9.22 years and 67.4% were male, in mild to moderately reduced EF group (35% ≤ EF < 50%), the mean of age was 60.89 ± 9.62 years, and 75.6% were males. In severely reduced EF patients (EF < 35%), the mean age was 61.05 ± 9.73 years and 80.2% of this group was male. In regard of risk factors, hypertension, dyslipidemia, obesity and family history were higher in normal EF group; while, diabetes mellitus, opium consumption and cigarette smoking were higher in severely reduced EF group. The proportion of performed off-pump surgery was higher in normal EF group (Table 1).

**Table 1:** Patients’ baseline characteristics based on pre-operative left ventricular ejection fraction

Ejection fraction (EF)	EF ≥ 50%	35% ≤ EF < 50%	EF < 35%	P value
Graft number	3 [3, 4]	4 [3, 4]	4 [3, 4]	< 0.001
Age (years)	60.75 ± 9.22	60.89 ± 9.62	61.05 ± 9.73	0.255
Gender				
Female	2453 (32.6%)	2544 (24.4%)	741 (19.8%)	< 0.001
Male	5073 (67.4%)	7890 (75.6%)	3002 (80.2%)	
eGFR (ml/min)				
≥ 90	2965 (47.3%)	3963 (44.7%)	1233 (37.4%)	< 0.001
60–89	2412 (38.5%)	3434 (38.7%)	1300 (39.5%)	
< 60	891 (14.2%)	1466 (16.5%)	761 (23.1%)	
Hypertension	4349 (57.8%)	5483 (52.6%)	1778 (47.6%)	< 0.001
Diabetes mellitus	2777 (36.9%)	4023 (38.6%)	1641 (43.9%)	< 0.001
Dyslipidemia	4561 (60.7%)	5678 (54.5%)	1804 (48.3%)	< 0.001
Positive Family History	3124 (41.5%)	3921 (37.6%)	1191 (31.8%)	< 0.001
Opium	824 (11.2%)	1472 (14.5%)	662 (18.3%)	< 0.001
Current cigarette smoker	1125 15.0%	1884 18.1%	778 20.9%	< 0.001
LM stenosis > 50%	669 (8.9%)	820 (7.9%)	364 (9.7%)	0.001
Pre-Surgery PCI	438 (5.8%)	731 (7.0%)	314 (8.4%)	< 0.001
Renal failure	128 (1.7%)	234 (2.3%)	131 (3.5%)	< 0.001
BMI ≥ 30 (kg/m^2^)	1968 (26.3%)	2459 (23.6%)	696 (18.7%)	< 0.001
Urgent/Emergent procedure	250 (3.3%)	405 (3.9%)	139 (3.7%)	0.139
COPD	192 (2.6%)	306 (3.0%)	144 (3.9%)	0.001
PVD	234 (3.2%)	229 (2.2%)	72 (2.0%)	0.124
Carotid artery stenosis				
20–50%	50 (0.7%)	55 (0.5%)	13 (0.4%)	0.144
51–75%	29 (0.4%)	35 (0.3%)	11 (0.3%)	
> 75%	81 (1.1%)	93 (0.9%)	26 (0.7%)	
CVA	388 (5.2%)	655 (6.3%)	296 (7.9%)	< 0.001
Pre CABG-MI Interval				
No MI	6136 (81.5%)	6522 (62.5%)	1663 (44.4%)	< 0.001
≤ 7Day	365 (4.8%)	992 (9.5%)	415 (11.1%)	
8–21 day	184 (2.4%)	622 (6.0%)	367 (9.8%)	
> 21Day	841 (11.2%)	2298 (22.0%)	1298 (34.7%)	
Off-pump surgery	858 (11.9%)	741 (7.3%)	293 (8.0%)	< 0.001

### Mid-term outcomes in EF groups

#### All-cause mortality

**Fig. 2:**
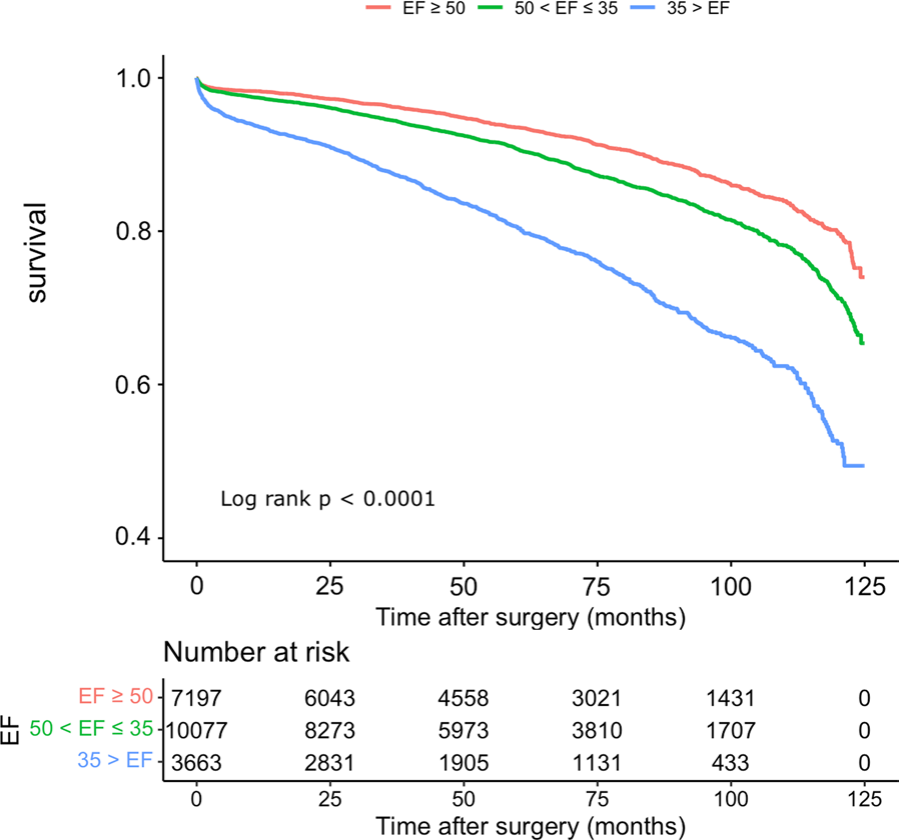
The Kaplan–Meier survival analysis of patients in three EF groups

Survival probability in patients with severe LV dysfunction (EF < 35%) is lower than the other two groups. This trend of lower survival becomes more significant in a longer follow-up duration (Fig. 2). The mortality rate in patients with normal EF, mild-moderately reduced EF and severely reduced EF were 9.5%, 12.8%, and 22.7%, respectively.

#### Non-fatal CCVE

**Fig. 3:**
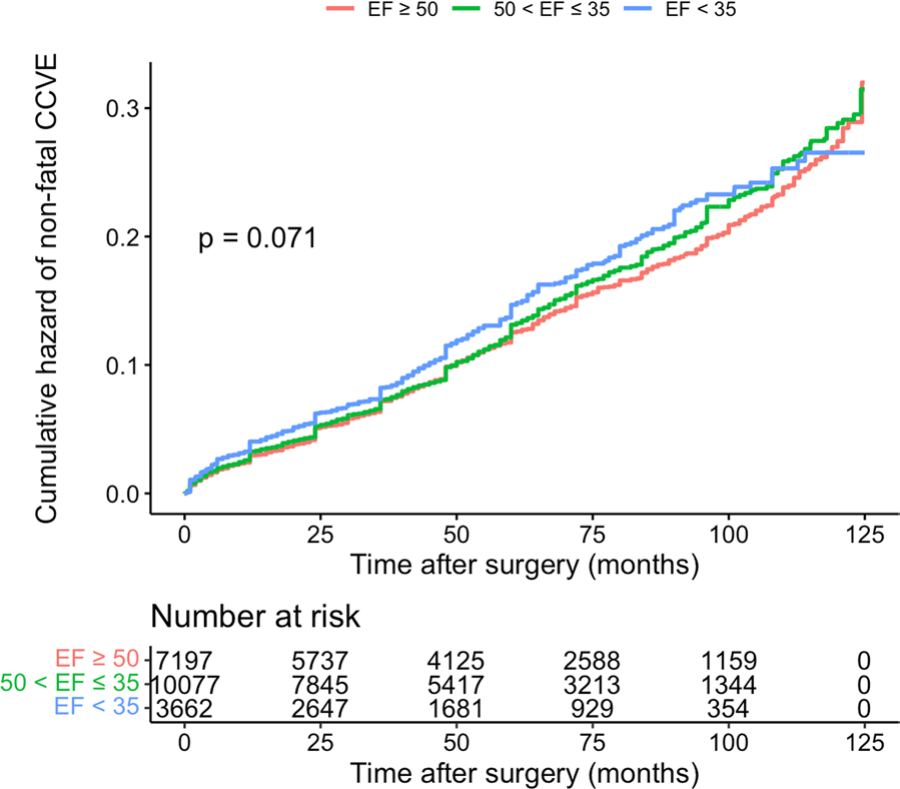
Cumulative hazard of non-fatal CCVE in three EF groups

Although the trend was higher in severe LV dysfunction, it was not significantly different between the three groups in survival analysis (*p* = 0.071) (Fig. 3). In normal EF, mild-moderately reduced EF, and severely reduced EF patients, the non-fatal CCVE rate were as follows; 12.7%, 12.6%, and 12%, respectively.

### Estimated HRs for predictors of mid-term outcomes

All univariate and multivariate cox regression analyses were reported in supplementary data (Additional file 1: Table S1).

#### All-cause mortality predictors

Graphical assessment for proportional hazard assumption could be found in supplementary (Additional file 1: Figure S1 and Additional file 1: Fig. S2).

In patients with EF ≥ 50%; age, diabetes mellitus, hypertension, estimated glomerular filtration rate (eGFR) < 60 ml/min, cerebrovascular accident (CVA), peripheral vascular disease (PVD), and carotid artery stenosis > 70% were significantly related to 5-year mortality.

In patients with mild-moderately reduced EF (50% < EF ≤ 35%); age, anemia, diabetes mellitus, hypertension, eGFR < 60 ml/min, CVA, COPD, left main (LM) stenosis > 50%, PVD, and carotid artery stenosis > 70% were estimated as risk factors (Table 2).

**Table 2:** Predictors of long-term Mortality: Final step of multivariable cox proportional stepwise regression analysis

All-cause mortality	Variable	Multivariate HR [95% CI]	*P* value
EF ≥ 50%	Age * (years)	1.047 [1.038, 1.056]	< 0.001
	Diabetes mellitus	1.519 [1.336, 1.727]	0.001
	Hypertension	1.502 [1.281, 1.762]	< 0.001
	eGFR < 60^†^ (ml/min)	1.390 [1.099 – 1.759]	0.006
	Dyslipidemia	0.783 [0.687, 0.893]	0.001
	CVA	1.467 [1.130, 1.904]	0.004
	PVD	1.687 [1.215, 2.342]	0.002
	Carotid stenosis > 70%	2.365 [1.472, 3.799]	< 0.001
50% < EF ≤ 35%	Age* (years)	1.031 [1.023, 1.038]	< 0.001
	Anemia	1.323 [1.123, 1.480]	0.001
	Positive family history	0.857 [0.759, 0.967]	0.012
	Diabetes mellitus	1.429 [1.275, 1.602]	< 0.001
	Hypertension	1.372 [1.195, 1.660]	< 0.001
	eGFR < 60^†^ (ml/min)	1.993 [1.663, 2.388]	< 0.001
	Dyslipidemia	0.887 [0.791, 0.995]	0.041
	LM stenosis > 50%	1.289 [1.123, 1.588]	0.015
	CVA	1.759 [1.468, 2.108]	< 0.001
	COPD	2.020 [1.606, 2.542]	< 0.001
	Off-pump surgery	0.697 [0.564, 0.861]	0.001
	PVD	1.477 [1.100, 1.981]	0.009
	Carotid stenosis > 70%	1.957 [1.285, 2.979]	0.002
EF < 35%	eGFR < 60^†^ (m/min)	1.825 1.399, 2.381]	< 0.001
	COPD	1.436 [1.027, 2.007]	0.034
	Graft number^‡^	0.889 [0.807, 0.978]	0.016

*HR estimated for increasing 1 year of age

^†^reference: eGFR > 90

^‡^HR estimated for increasing 1 graft

In patients with severely reduced EF (EF < 35%); COPD and eGFR < 60 ml/min were revealed as potential mortality risk factors.

#### Non-fatal CCVEs predictors

For patients with normal EF (EF ≥ 50%); female gender, diabetes mellitus, hypertension, CVA, pre-surgery PCI, and positive family history were shown to be significant.

In mild to moderately reduced EF patients (50% < EF ≤ 35%); female gender, diabetes mellitus, hypertension, CVA, cigarette smoking, pre-surgery PCI, and positive family history were associated with a higher risk of non-fatal CCVEs. For severe reduced EF patients (EF < 35%); diabetes mellitus, and eGFR < 90 ml/min were related to higher non-fatal CCVEs occurrence (Table 3).

**Table 3:** Predictors of non-fatal CCVEs: Final step of multivariable cox proportional stepwise regression analysis

Non-fatal CCVEs	Variable	Multivariate HR [95% CI]	*P* value
EF ≥ 50%	Female	1.359 [1.189, 1.554]	< 0.001
	CVA	1.524 [1.181, 1.967]	0.001
	Pre-Surgery PCI	1.611 [1.268, 2.046]	< 0.001
	Positive Family History	1.208 [1.067, 1.369]	0.003
	Hypertension	1.206 [1.055, 1.378]	0.006
	Diabetes mellitus	1.156 [1.014, 1.318]	0.030
50% < EF ≤ 35%	Female	1.287 [1.135, 1.459]	< 0.001
	Pre-Surgery PCI	1.401 [1.146, 1.713]	0.001
	Diabetes mellitus	1.190 [1.064, 1.331]	0.002
	Hypertension	1.226 [1.096, 1.372]	< 0.001
	CVA	1.513 [1.224, 1.870]	< 0.001
	Current cigarette smoker	1.235 [1.069, 1.427]	0.004
	Positive Family History	1.233 [1.002, 1.518]	0.048
EF < 35%	Diabetes mellitus	1.284 [1.106, 1.491]	< 0.001
	60 < eGFR < 90^†^ (ml/min)	1.317 [1.045, 1.659]	0.020
	eGFR < 60^†^ (ml/min)	1.456 [1.114, 1.904]	0.006

^†^Reference: eGFR > 90 (ml/min)

## Discussion

Based on the results of this large sample size registry-based cohort study, distribution of risk factors and predictors of mortality and non-fatal CCVE were not the same in each EF group but had many points in common. Diabetes mellitus is the common risk factor of mortality and CCVE in almost all EF groups. Impaired renal function (eGFR < 60 ml/min) was associated with a higher risk of mortality after CABG regardless of EF level. Older age, diabetes mellitus, history of CVA, and COPD were associated with a higher risk of mortality in the EF < 50% group (both mild to moderate and severely reduced groups). Carotid artery stenosis > 70% was associated with increased risk of mortality in patients with normal and mild to moderate reduced EF.

Although the mortality rate was significantly higher in the severely reduced EF group, this was not statistically significant for non-fatal CCVE. Similar to our findings, Maltais et al. evaluated 1250 patients who underwent off-pump CABG and showed that major adverse cardiac events (MACEs) were not significantly different in patients with LVEF < 35% and LVEF ≥ 35% after adjustment for potential risk factors [20]. However, another study conducted by El-Shafey et al. evaluated 170 patients who underwent CABG and showed that non-fatal CCVE occurred more significantly in patients with LVEF < 40% [21].

Another important and noticeable finding was the role of the female gender in post-CABG outcomes. Although female gender was not significantly associated with higher mortality, it was associated with higher non-fatal CCVE in patients with normal and mild to moderately reduced EF. Similar to our findings, Kurlansky et al. evaluated all patients who underwent coronary revascularization and found that outcomes (MACEs and all-cause mortality) were worse in women who underwent either CABG or PCI [22]. Also, Huckaby et al. evaluated 6163 patients undergoing coronary revascularization and showed that 1-year outcomes (MACE and death) were worse among women with multivessel disease who underwent either CABG or PCI [23]. Besides, a meta-analysis of 20 studies showed that women had an increased risk of short-, mid-, and long-term mortality after isolated CABG compared to men [24]. Another study conducted by Ergunes et al. found that the in-hospital mortality rate was higher in female patients however, the mid-term survival was similar between males and females [25]. However, according to our results, the female gender was not associated with an increased risk of mortality.

Patients with impaired LVEF and CAD have multiple hemodynamic and metabolic abnormalities at rest such as altered myocardial oxygen consumption and lactate metabolism [26]. Therefore, patients with low EF who undergo CABG are a distinctive group of patients and may have different risk factors associated with postoperative outcomes compared to those with normal EF [3]. Therefore, identification of risk factors associated with adverse outcomes after CABG and selection of patients is important for achieving the optimal postoperative outcome.

According to the surgical treatment for ischemic heart failure extension (STICHES) trial, CABG had clear survival benefits over medical therapy in patients with LVEF < 35% at 10-year follow-up [8]. Although CABG is superior to medical therapy in terms of better survival, the outcomes of patients with low LVEF were shown to be worse compared to those with normal EF [10]. Besides the role of net EF value in the post-op outcome, other preoperative predictors also play an important role here. As mentioned before, the distribution and the strength of these predictors are different in each EF group.

Topkara et al. [11] analyzed 55,515 patients who underwent CABG and showed that independent predictors of in-hospital mortality in patients with EF ≤ 20% are older age, female gender, renal failure, previous myocardial infarction (< 6 h), and previous open-heart operation. According to our results, older age was the independent predictor of all-cause mortality in patients with EF < 50, and female gender was the independent predictor of non-fatal CCVEs in patients with reduced EF (35% ≤ EF < 50%). Shapira et al. [27] evaluated 115 patients with EF ≤ 30% who underwent isolated CABG. They found that female gender, renal failure, respiratory complications, and mitral regurgitation are independent predictors of mid-term (36 months) mortality in these patients’ groups. Kamal et al. [12] evaluated two propensity-score matched groups (EF < 50% and EF ≥ 50%) who underwent isolated CABG. They showed that the use of an intra-aortic balloon pump was the independent predictor of early mortality in patients with EF < 50%. Soliman Hamad et al. [3] assessed 413 patients with EF ≤ 30% who underwent isolated CABG. They found that age, hemoglobin levels, and creatinine levels are predictors of early mortality after CABG. Vickneson et al. [28] analyzed CABG results of 346 patients with EF ≤ 30% and found that hemodynamic instability and kidney dysfunction are independent predictors of 30-day mortality. Similarly, we showed that anemia and eGFR < 60 ml/min are independent predictors of all-cause mortality in patients with reduced EF (35% ≤ EF < 50%). Khaled et al. [29] evaluated 110 patients with EF < 50% who underwent CABG. They showed that diabetes mellitus, diastolic dysfunction, and the use of intra-aortic balloon pumps were predictors of mortality in the study population. Similarly, we found that diabetes mellitus was the independent predictor of all-cause mortality and non-fatal CCVEs in patients with EF < 50%. Higher rates of adverse outcomes in patients with diabetes mellitus may be due to adverse effects of insulin therapy, inflammatory response, and hormonal overreaction which leads to disruption of cardiovascular function [30]. Gatti et al. [31] conducted a study of 300 patients with EF ≤ 35% and showed that poor glycemic control and GFR < 50 ml/min were independent risk factors for in-hospital mortality.

According to our results, dyslipidemia and positive family history were protective factors for all-cause mortality in the reduced EF group. This observation could be partly explained by the utilization of cardiovascular medications such as aspirin, beta-blockers, and statins in patients with a family history of coronary disease [32]. Moreover, they are more likely to exercise, have a healthy diet, be aware of cardiovascular risks, and manage modifiable risk factors such as hypertension [33, 34]. Also, patients with dyslipidemia are more likely to use lipid-lowering medications such as statins. It has been shown that statin therapy is associated with a lower risk of all-cause mortality and MACE after CABG [35, 36].

Similarly, Abdi-Ali et al. [32] reported that in patients with proven coronary disease, positive family history was associated with a 23% relative risk reduction of all-cause mortality over 5.6 years. Two other studies conducted by Canto et al. [37] and Agarwal et al. [38] showed that in a large population of patients with acute myocardial infarction, positive family history is associated with lower in-hospital mortality.

### Strength and limitation

The present study should be interpreted in the context of several possible limitations. Our findings were based on midterm follow-up (median 5.61 years), and further studies with longer follow-up are needed to achieve more accurate results. This study was conducted in a single center and the generalizability of our results should be assessed. Still, THC is a referral educational university that serves patients from all over the country. We did not have or permission to have the ‘’autopsy’’ report of the patients hence the cause of death was unclear.

The major strengths of this study are as follows; First, large sample size presented a considerably high prevalence of events which enhances the power of the study; Second, our data extracted from THC registry data bank which records patient’s data prospectively; Third, to overcome surgical expertise limitation, we chose expert surgeons which conducted at least 100 off-pump and 400 on-pump CABG procedures previously.

## Conclusion

Patients with severely reduced EF are at higher risk of mortality after CABG however the rate of events may not be necessarily higher after adjustment for multiple pre-operative comorbidities.

Diabetes mellitus and impaired renal function are important mortality predictors regardless of EF level.

## Supplementary Information

**Additional file 1**. **Table S1:** Univariate and multivariate cox regression analyses; **Figure S1 and Figure S2:** Graphical assessment for proportional hazard assumption. 

## Data Availability

The datasets used and/or analyzed during the current study are available from the corresponding author on reasonable request.

## Abbreviations

EF: Ejection Fraction
PCI: Percutaneous Coronary Intervention
CABG: Coronary Artery Bypass Graft
MACE: Major Adverse Cardiac Event
CCVEs: Cerebro-CardioVascular Events
COPD: Chronic Obstructive Pulmonary Disease
LVEF: Left Ventricular Ejection Fraction
LIMA and RIMA: Left and Right Internal Mammillary Artery
SVG: Saphenous Vein Graft
CPB: CardioPulmonary Bypass
GFR: Glomerular Filtration Rate
ACT: Activated Clotting Time\
ACS: Acute Coronary Syndrome
BMI: Body mass index
Hb: Hemoglobin
eGFR: Estimated glomerular filtration rate
PCI: Percutaneous coronary intervention
COPD: Chronic obstructive pulmonary disease
CVA: Cerebrovascular accident
MI: Myocardial infarction
