# Supplementary material for: Significance of preoperative left ventricular ejection fraction in 5-year outcome after isolated CABG
Source: J Cardiothorac Surg. 2021 Dec 27;16:353. doi: 10.1186/s13019-021-01732-3 (PMC8711149; doi:10.1186/s13019-021-01732-3)
Supplement: Supplementary file 1 — Additional file 1. Table S1: Univariate and multivariate cox regression analyses; Figure S1 and Figure S2: Graphical assessment for proportional hazard assumption. [file 13019_2021_1732_MOESM1_ESM.docx]

**Definition of Variables**

The following data were included for analysis: demographic characteristics, graft numbers, comorbidities, preoperative risk factors, and urgency of surgery.

Diabetes mellitus was defined as fasting plasma glucose ≥ 126 mg/dL and/or random plasma glucose ≥ 200 mg/dL and/or hemoglobin A1c (HbA1c) ≥ 6.5% (13) and/or treatment with either oral hypoglycemic agents or insulin. Minimum systolic blood pressure of 140 mm Hg or a minimum diastolic blood pressure of 90 mm Hg or a history of antihypertensive therapy labeled as hypertension. Dyslipidemia considers as existence one of follows, minimum total cholesterol level of 240 mg/dL; LDL-C level more than 160 mg/dL; a minimum triglyceride level of 200 mg/dL; and HDL-C of less than 40 mg/dL in men and less than 50 mg/dL in women; or a history of prescribed lipid medications based on the National Cholesterol Education Program (NCEP) Adult Treatment Plan (ATP) III (14). Renal failure was defined as glomerular filtration rate<60 mL/min/1.73 m^2^ or stage 3a and higher chronic kidney disease. GFR was estimated by the Cockcroft-Gault formula. Cerebrovascular accident was defined as, ischemic stroke, hemorrhagic stroke, and transient ischemic attack. A family history of CAD was defined as having a first-degree relative with a history of CAD; i.e., acute myocardial infarction or documented CAD which diagnosed by either invasive coronary angiography or computed tomography coronary angiography. Cigarette smoking status was defined as current smoker and stated from the patient’s self-reported status. Opium consumption was defined as the current consumption of opium either smoking opium or drinking opium dissolved in tea. Patients divided into three categories in the term of urgency of the procedure, emergent (surgery should take place as soon as possible, in the following 6 hours), urgent (surgery should take place in the following 6-24 hours), and elective.

| Table 1. Estimated HRs | | Mortality | | | Non-fatal cardiovascular events | | |
| --- | --- | --- | --- | --- | --- | --- | --- |
|  |  | EF ≥ 50% | 50 < EF ≤ 35 | 35 > EF | EF ≥ 50% | 50 < EF ≤ 35 | 35 > EF |
| Female | | 1.108 [0.949, 1.293] | 1.019 [0.876, 1.184] | 1.126 [0.956, 1.328] | 1.359 [1.189, 1.554] | 1.287 [1.135, 1.459] | 1.176 [0.944, 1.466] |
| Age* | | 1.047 [1.038, 1.056] | 1.031 [1.023, 1.038] | 1.003 [0.988, 1.019] | 0.997 [0.991, 1.004] | 0.991 [0.985, 1.001] | 0.999 [0.990, 1.009] |
| Anemia | | 1.132 [0.940, 1.364] | 1.323 [1.123, 1.480] | 1.170 [0.999, 1.370] | 1.056 [0.897, 1.245] | 1.021 [0.897, 1.162] | 1.069 [0.870, 1.313] |
| eGFR† | >=90 | Reference | Reference | Reference | Reference | Reference | Reference |
|  | 60-89 | 0.900 [0.748, 1.084] | 1.140 [0.965, 1.348] | 1.126 0.854, 1.485] | 1.033 [0.885, 1.204] | 1.115 [0.968, 1.284] | 1.317 [1.045, 1.659] |
|  | <60 | 1.390 [1.099 – 1.759] | 1.993 [1.663, 2.388] | 1.825 1.399, 2.381] | 1.004 [0.808, 1.249] | 1.190 [1.064, 1.331] | 1.456 [1.114, 1.904] |
| Hypertension | | 1.502 [1.281, 1.762] | 1.372 [1.195, 1.660] | 1.116 [0.958, 1.300] | 1.206 [1.055, 1.378] | 1.226 [1.096, 1.372] | 1.076 [0.896, 1.292] |
| BMI=>30 | | 0.883 [0.742, 1.050] | 0.960 [0.843, 1.093] | 0.869 [0.726, 1.040] | 1.134 [0.987, 1.304] | 1.098 [0.948, 1.273] | 1.074 [0.855, 1.350] |
| Diabetes | | 1.519 [1.336, 1.727] | 1.429 [1.275, 1.602] | 1.124 [0.929, 1.361] | 1.156 [1.014, 1.318] | 1.190 [1.064, 1.331] | 1.102 [0.917, 1.324] |
| Dyslipidemia | | 0.783 [0.687, 0.893] | 0.887 [0.791, 0.995] | 0.889 [0.762, 1.037] | 1.105 [0.961, 1.264] | 0.994 [0.873, 1.133] | 1.027 [0.855, 1.233] |
| Current cigarette smoker | | 1.170 [0.954, 1.434] | 1.009 [0.901, 1.129] | 0.854 [0.713, 1.022] | 0.944 [0.787, 1.132] | 1.235 [1.069, 1.427] | 0.908 [0.716, 1.157] |
| Opium | | 1.111 [0.872, 1.048] | 1.038 [0.881, 1.224] | 1.121 [0.974, 1.290] | 1.074 [0.876, 1.316] | 1.031 [0.879, 1.210] | 1.093 [0.853, 1.401] |
| COPD | | 1.156 [0.720, 1.856] | 2.020 [1.606, 2.542] | 1.436 [1.027, 2.007] | 1.384 [0.943, 2.032] | 0.988 [0.688, 1.420] | 1.142 [0.693, 1.882] |
| Cerebrovascular accident | | 1.467 [1.130, 1.904] | 1.759 [1.468, 2.108] | 1.196 [0.819, 1.747] | 1.524 [1.181, 1.967] | 1.513 [1.224, 1.870] | 1.187 [0.840, 1.677] |
| Positive Family History | | 0.871 [0.723, 1.048] | 0.857 [0.759, 0.967] | 0.931 [0.786, 1.103] | 1.208 [1.067, 1.369] | 1.023 [0.918, 1.140] | 1.233 [1.002, 1.518] |
| LM stenosis > 50% | | 1.222 [0.943, 1.584] | 1.289 [1.123, 1.588] | 1.129 [0.891, 1.431] | 1.079 [0.862, 1.351] | 1.009 [0.818, 1.244] | 0.950 [0.684, 1.319] |
| Graft number‡ | | 0.952 [0.878, 1.032] | 0.985 [0.925, 1.049] | 0.889 [0.807, 0.978] | 0.944 [0.881, 1.011] | 0.973 [0.915, 1.035] | 0.956 [0.846, 1.059] |
| Pre-Surgery PCI | | 1.225 [0.844, 1.777] | 1.075 [0.851, 1.358] | 1.064 [0.814, 1.390] | 1.611 [1.268, 2.046] | 1.401 [1.146, 1.713] | 1.330 [0.954, 1.855] |
| Urgent/Emergent procedure | | 0.999 [0.593, 1.684] | 0.825 [0.647, 1.051] | 1.033 [0.775, 1.378] | 1.109 [0.829, 1.483] | 1.133 [0.907, 1.414] | 0.819 [0.522, 1.283] |
| Off-pump surgery | | 1.176 [0.868, 1.593] | 0.697 [0.564, 0.861] | 0.829 [0.607, 1.133] | 0.964 [0.787, 1.180] | 0.855 [0.695, 1.054] | 0.941 [0.548, 1.616] |
| PVD | | 1.687 [1.215, 2.342] | 1.477 [1.100, 1.981] | 1.078 [0.644, 1.804] | 1.079 [0.780, 1.493] | 1.182 [0.864, 1.617] | 0.695 [0.221, 2.181] |
| Carotid stenosis | No stenosis | Reference | Reference | Reference | Reference | Reference | Reference |
|  | 20%-50% | 0.314 [.078, 1.260] | 1.079 [0.655, 1.777] | 0.419 [0.058, 3.055] | 0.783 [0.351, 1.747] | 0.889 [0.443, 1.782] | 0.924 [0.221, 3.248] |
|  | 51%-75% | 1.684 [0.628, 4.518] | 1.297 [0.535, 3.146] | 0.821 [0.123, 3.445] | 0.398 [0.056, 2.828] | 1.418 [0.531, 3.787] | 1.234 [0.123, 5.214] |
|  | >75% | 2.365 [1.472, 3.799] | 1.957 [1.285, 2.979] | 0.967 [0,303 3.089] | 0.933 [0.444, 1.962] | 0.603 [0.270, 1.345] | 1.458 [0.221, 6.879] |

*HR estimated for increasing 1 year of age, † reference: eGFR > 90, ‡ HR estimated for increasing 1 graft

| 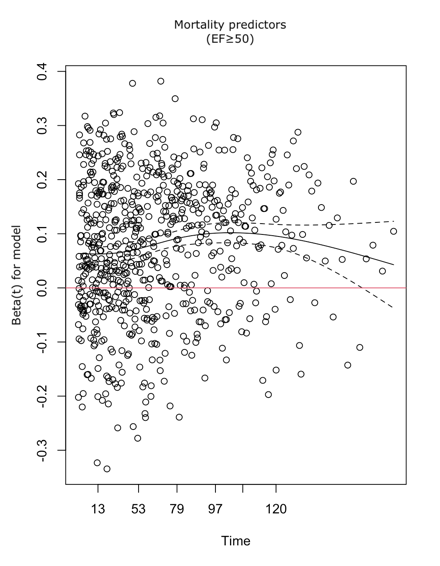 | 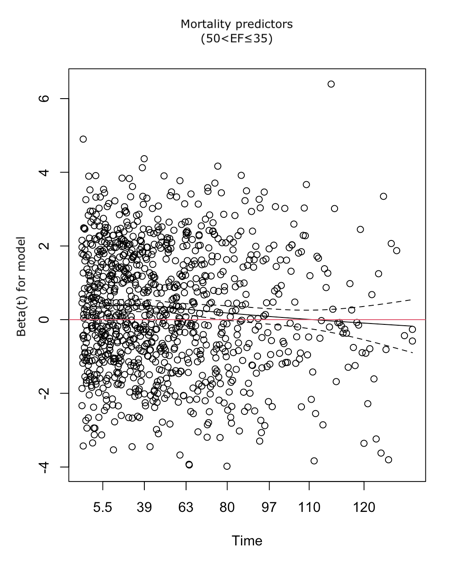 | 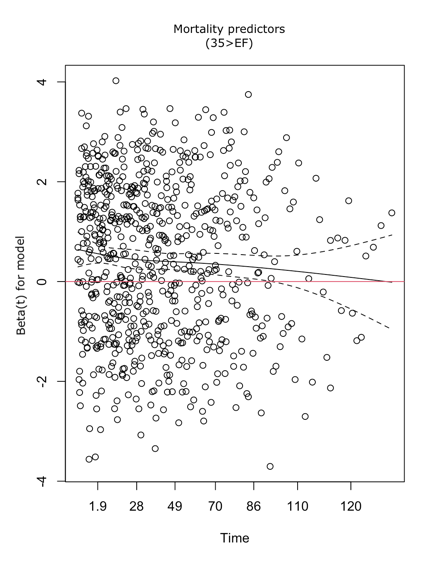 |
| --- | --- | --- |
| A. EF ≥ 50 | B. 50 < EF ≤ 35 | C. 35 > EF |

Figure 1. Graphical assessment for proportional hazard assumption (Mortality predictors)

| 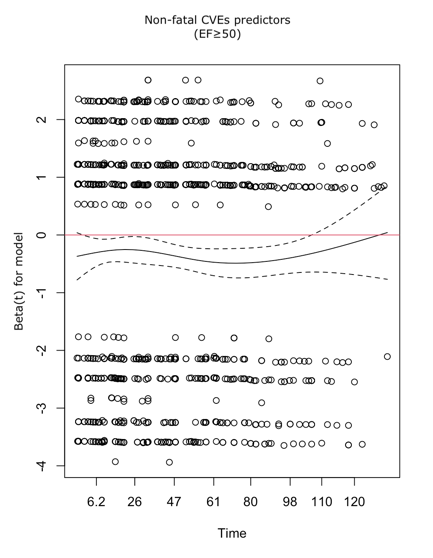 | 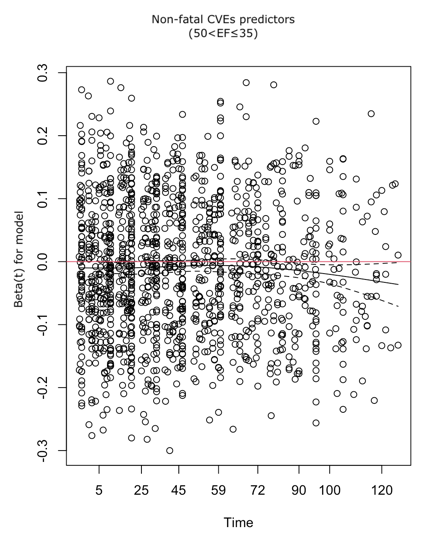 | 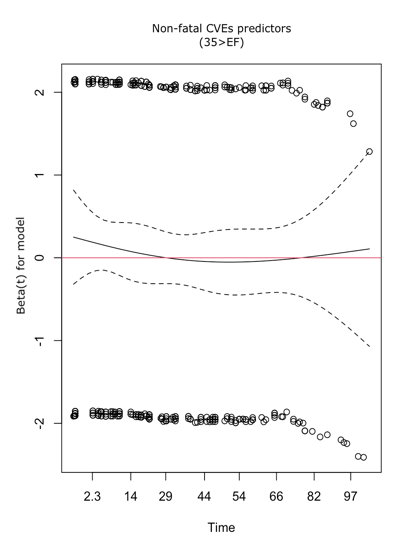 |
| --- | --- | --- |
| A. EF ≥ 50 | B. 50 < EF ≤ 35 | C. 35 > EF |

Figure 2. Graphical assessment for proportional hazard assumption (Non-fatal CCVEs predictors)
